# In memoriam of Professor Gregory Schultz (1948–2024)

**DOI:** 10.1111/iwj.14896

**Published:** 2024-04-29

**Authors:** Keith Harding, Douglas Queen

**Affiliations:** ^1^ Editor‐in‐Chief IWJ Cardiff United Kingdom; ^2^ Editor IWJ Toronto Ontario Canada

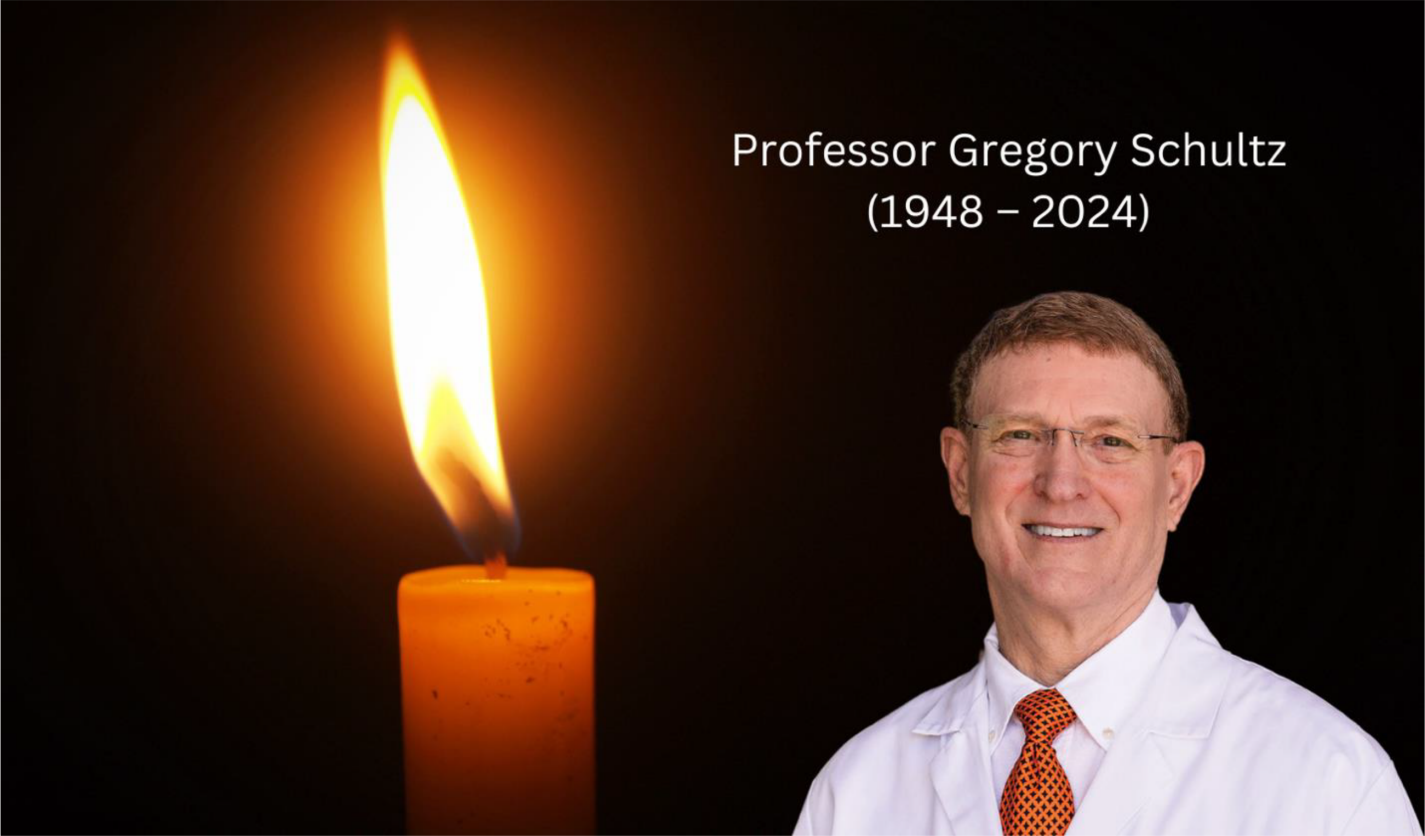

The International Wound Journal family is deeply saddened to hear of the passing of one of its founding editorial board members and a wound care legend. There are few in the wound care world that do not know the name Greg Schultz. His contribution to our field is beyond significant and will continue to inspire other researchers beyond his life. Wound care has lost one of its true gentlemen.

As a long‐standing wound researcher, Greg contributed significantly to the world of wound care, which included the IWJ. Gregory Schultz, PhD, was until recently the Professor of Obstetrics and Gynecology and Director of the Institute for Wound Research at the University of Florida. His journey with science started with a BS and PhD degree in Biochemistry, with his postdoctoral work leading him to Yale and cell biology. But we all know him for his research focus on the role that bacterial biofilms play in stimulating chronic inflammation that leads to elevated levels of proteases in wounds.

Professor Schultz authored and co‐authored over 420 publications which have been cited over 26,000 times. He received over $40 million in grant support for numerous studies and is an inventor on over 36 patents. He co‐founded two successful biotechnology companies, being elected Fellow of the National Academy of Inventors in 2021.

He served as President of the Wound Healing Society (1999–2001), a member of the National Pressure Ulcer Advisory Panel (2007–2010) and a member of the Wound Healing Foundation Board of Directors (2016–present), to name only a few of the many societies he was involved with during his life.

Greg was until his untimely death a member of our editorial board from day one of the creation of the International Wound Journal. Here are some of his contributions to our journal in the 20 years of its existence:

Schultz GS, Barillo DJ, Mozingo DW and Chin GA. Wound bed preparation and a brief history of TIME. *Int Wound J*. 2004;1:19–32. https://doi.org/10.1111/j.1742-481x.2004.00008.x

Acosta JB, Garcia del Barco D, Cibrian Vera D, Savigne W, Lopez‐Saura P, Guillen Nieto G and Schultz GS. The pro‐inflammatory environment in recalcitrant diabetic foot wounds. *Int Wound J*. 2008;5:530–539. https://doi.org/10.1111/j.1742-481X.2008.00457.x

Baskovich B, Sampson EM, Schultz GS and Parnell LK. Wound dressing components degrade proteins detrimental to wound healing. *Int Wound J*. 2008;5:543–551. https://doi.org/10.1111/j.1742-481X.2007.00422.x

Leaper DJ, Schultz G, Carville K, Fletcher J, Swanson T and Drake R. Extending the TIME concept: what have we learned in the past 10 years?†. *Int Wound J*. 2012;9:1–19. https://doi.org/10.1111/j.1742-481X.2012.01097.x

Phillips PL, Yang Q and Schultz GS. The effect of negative pressure wound therapy with periodic instillation using antimicrobial solutions on *Pseudomonas aeruginosa* biofilm on porcine skin explants. *Int Wound J*. 2013;10:48–55. https://doi.org/10.1111/iwj.12180

Phillips PL, Yang Q, Davis S, Sampson EM, Azeke JI, Hamad A and Schultz GS. Antimicrobial dressing efficacy against mature Pseudomonas aeruginosa biofilm on porcine skin explants. *Int Wound J*. 2015;12:469–483. https://doi.org/10.1111/iwj.12142

Gowda S, Weinstein DA, Blalock TD, Gandhi K, Mast BA, Chin G and Schultz GS. Topical application of recombinant platelet‐derived growth factor increases the rate of healing and the level of proteins that regulate this response. *Int Wound J*. 2015;12:564–571. https://doi.org/10.1111/iwj.12165

Jeong S, Schultz GS and Gibson DJ. Testing the influence of surfactant‐based wound dressings on proteinase activity. *Int Wound J*. 2017;14:786–790. https://doi.org/10.1111/iwj.12697

Yang Q, Larose C, Della Porta AC, Schultz GS and Gibson DJ. A surfactant‐based wound dressing can reduce bacterial biofilms in a porcine skin explant model. *Int Wound J*. 2017;14:408–413. https://doi.org/10.1111/iwj.12619

Roche ED, Woodmansey EJ, Yang Q, Gibson DJ, Zhang H, Schultz GS. Cadexomer iodine effectively reduces bacterial biofilm in porcine wounds ex vivo and in vivo. *Int Wound J*. 2019;16:674–683. https://doi.org/10.1111/iwj.13080

Personal reflection (DQ): In my 35+ years in this area, I had the honour of meeting Greg many times. Each time was insightful but also enjoyable. Greg knew how to engage and inform regardless of your background and experience. In 2008 at the World Union meeting in Toronto, Greg was awarded a lifetime achievement award. Witnessing him receiving this award among the ‘best of the best’ in wound care cemented his ‘rock star’ status in my mind, regardless of the fact he went on to contribute for a further nearly two decades.

Personal reflection (KGH)—Greg was an outstanding scientist and educator—one of the best. As a global leader in research into the microbiology of wounds and an excellent and inspiring educator, he always made time to help others with their research. A major area of his research focused on defining the role of bacterial biofilms in stimulating chronic inflammation and proteases that impair healing in chronic wounds. He made the biology of wound healing understandable and interesting to the practicing clinician. He was passionate about the science behind healing and supported many students at various centres around the World.

Greg was a true gentleman and someone who I worked with on many occasions—he will be missed moving forward. He always had time to talk and put you in contact with people. He was admired and respected by all he met. I was fortunate to have the honour to publish with Greg as a co‐author and without doubt it was one of the privileges and highlights of my academic career.

His passing while sad reminds us all of the extensive influence of Professor Gregory S. Schultz in the wound care area and he will leave a legacy for other clinicians and scientists to benefit from. His legacy will live on through the continued work of many others.

